# Discovery of *CDH23* as a Significant Contributor to Progressive Postlingual Sensorineural Hearing Loss in Koreans

**DOI:** 10.1371/journal.pone.0165680

**Published:** 2016-10-28

**Authors:** Bong Jik Kim, Ah Reum Kim, Chung Lee, So Young Kim, Nayoung K. D. Kim, Mun Young Chang, Jihye Rhee, Mi-Hyun Park, Soo Kyung Koo, Min Young Kim, Jin Hee Han, Seung-ha Oh, Woong-Yang Park, Byung Yoon Choi

**Affiliations:** 1 Department of Otorhinolaryngology, Seoul National University Hospital, Seoul National University College of Medicine, Seoul, Korea; 2 Samsung Genome Institute, Samsung Medical Center, Seoul, Korea; 3 Department of Health Sciences and Technology, SAIHST, Sungkyunkwan University, Suwon, Korea; 4 Division of Intractable Diseases, Center for Biomedical Sciences, National Institute of Health, Chungcheongbuk-do, Korea; 5 Department of Otorhinolaryngology, Seoul National University Bundang Hospital, Seongnam, Korea; 6 Department of Molecular Cell Biology, School of Medicine, Sungkyunkwan University, Suwon, Korea; McMaster University, CANADA

## Abstract

*CDH23* mutations have mostly been associated with prelingual severe-to-profound sensorineural hearing loss (SNHL) in either syndromic or nonsyndromic SNHL (DFNB12). Herein, we demonstrate the contribution of *CDH23* mutations to postlingual nonsyndromic SNHL (NS-SNHL). We screened 32 Korean adult probands with postlingual NS-SNHL sporadically or in autosomal recessive fashion using targeted panel or whole exome sequencing. We identified four (12.5%, 4/32) potential postlingual DFNB12 families that segregated the recessive *CDH23* variants, qualifying for our criteria along with rapidly progressive SNHL. Three of the four families carried one definite pathogenic *CDH23* variant previously known as the prelingual DFNB12 variant in a *trans* configuration with rare *CDH23* variants. To determine the contribution of rare *CDH23* variants to the postlingual NS-SNHL, we checked the minor allele frequency (MAF) of *CDH23* variants detected from our postlingual NS-SNHL cohort and prelingual NS-SNHL cohort, among the 2040 normal control chromosomes. The allele frequency of these *CDH23* variants in our postlingual cohort was 12.5%, which was significantly higher than that of the 2040 control chromosomes (5.53%), confirming the contribution of these rare *CDH23* variants to postlingual NS-SNHL. Furthermore, MAF of rare *CDH23* variants from the postlingual NS-SNHL group was significantly higher than that from the prelingual NS-SNHL group. This study demonstrates an important contribution of *CDH23* mutations to poslingual NS-SNHL and shows that the phenotypic spectrum of DFNB12 can be broadened even into the presbycusis, depending on the pathogenic potential of variants. We also propose that pathogenic potential of *CDH23* variants and the clinical fate of DFNB12 may be predicted by MAF.

## Introduction

Mutations of *CDH23* (NM_022124) have been associated with type 1D Usher Syndrome (USH1D) and nonsyndromic hearing loss (DFNB12), in a recessively transmitted manner [1, 2]. USH1D is associated with severe manifestations, including congenital profound deafness vestibular areflexia, and visual problems due to retinitis pigmentosa. Conversely, DFNB12, is characterized by prelingual-onset nonsyndromic sensorineural hearing loss (SNHL) without the impairment of vestibular or visual functions [3].

The importance of *CDH23* as a deafness gene has increasingly been recognized [4, 5]. In a Japanese study, *CDH23* mutations were reported to be frequent after *GJB*2 and *SLC26A4* in children and adults with hearing impairment [6]. Recently, *CDH23* mutations have been reported in the Korean deaf population [7, 8], and genetic loads of *CDH23* and its implications in the Korean pediatric population have also recently been reported [9]. Accordingly, p.P240L in *CDH23* proved to exert a strong founder effect in the Korean pediatric population with severe-to-profound nonsyndromic SNHL. Although *CDH23* has been well established as a deafness gene, it is challenging to delineate its function by a functional assay due to its relatively large size with 69 exons and encoded cadherin 23, which includes a protein of 3,354 amino acids with 27 extracellular cadherin (EC) domains, a single transmembrane domain, and a short cytoplasmic domain [4, 5, 10].

*CDH23* related hearing loss is known to be associated with its role in the tip links of the inner ear hair cells. Tip links are extracellular filaments that is proposed to act as a gate for the mechanotransduction channel. In other words, it transduces the mechanical forces that arise from the sound waves and head movement, allowing one to hear and maintain balance [11]. The interaction between protocadherin-15 (*PCDH15*) and *CDH23*—both localized in the upper and lower parts of the tip link complex—has been reported to form tip links [12]. Accordingly, both *CDH23* and *PCDH15* are necessary for normal mechanotransduction, and mutations in these genes have been associated with sensory impairment [10, 13].

*CDH23* related hearing loss in USH1D and DFNB12 has mostly been associated with either congenital or prelingual-onset hearing loss [5]. However, some *CDH23* mutations have been reported to be associated with postlingual-onset moderate hearing loss in humans [6, 14]. Furthermore, some *Cdh*23 mutant alleles in mice manifested age-related hearing loss, which started as high-frequency hearing loss that eventually progressed to profound impairment with varying degrees of rapidity, as explained by the allelism and modifier gene [15–17]. Moreover, *Cdh23* was also found to be susceptible to noise induced hearing loss, which is a different type of SNHL [18, 19]. However, the contribution of *CDH23* to the human postlingual-onset hearing loss has not been adequately investigated. Moreover, mechanisms responsible for different phenotypes of *CDH23* mutations have not been fully elucidated to date.

Herein, we adopted a genetic epidemiologic approach to objectively illustrate the importance of *CDH23* in postlingual-onset SNHL and to investigate the causal relationship between genotypes and phenotypes. In brief, we estimated the carrier frequency of *CDH23* mutations in postlingual adult-onset inherited hearing loss, which were segregated in either a sporadic or autosomal recessive (AR) fashion based on the ethnic-specific minor allele frequency (MAF) filtering process to investigate the contribution of *CDH23* and further tried to delineate the genotypic hierarchy of *CDH23* mutations that determine the fate of DFNB12.

## Material and Methods

### Ethical considerations

This study was approved by the Institutional Review Boards of Seoul National University Hospital (IRBY-H-0905-041-281) and Seoul National University Bundang Hospital (IRB-B-1007-105-402). Written informed consent was obtained from all study participants. In the case of minors, written informed consent was obtained from parents or guardians.

### Study participants and clinical data

Patients with postlingual adult-onset SNHL, segregated in either a sporadic or AR fashion, were selected from our Korean cohorts, and published data from a previous study on pediatric cohorts were retrieved for analysis [9]. Additionally, our adult cohort fulfilled the following criteria: 1) bilateral nonsyndromic hearing loss, 2) moderate hearing loss with progressive nature, and 3) onset of hearing loss at the age of 15 or older, excluding the possibility of later development of USH1D. Also, when possible, family members were invited to participate in the study. Clinical data were obtained for this study population, including gender, age, medical history, physical examination, and audiological test results. The hearing threshold was calculated by averaging the thresholds of 0.5, 1, 2 and 4 kHz, which was classified into five categories: subtle (16–25 dB), mild (26–40 dB), moderate (41–70 dB), severe (71–95 dB), and profound (>95 dB) [20].

### Molecular genetic diagnosis of postlingual SNHL

Genomic DNA was extracted from the peripheral blood samples or buccal cells, using the standard protocols (Gentra Puregene Blood Kit, Qiagen, cat. 158389; Venlo, Limburg, Netherlands). After *GJB2* sequencing, we performed targeted resequencing of the known 129,200 deafness genes (TRS-129 and TRS-200), as previously described [21, 22]. TRS-129 and TRS-200 were performed by Otogenetics (http://www.otogenetics.com/) and SGI (Samsung genomic institute, http://www.samsunghospital.com/dept/main/index.do?DP_CODE=BP7), respectively. The obtained reads were aligned to the UCSC hg19 reference genome (http://genome.ucsc.edu/index.html) and variants were filtered. Further bioinformatics analyses were performed as previously described [23]. If the results of these steps were not convincing, WES was performed. Thereafter, the final candidate variants in these families were verified by Sanger sequencing and validated by ethnic-specific MAF filtering in 200 unrelated Korean control chromosomes from 100 normal hearing control subjects. Pathogenicity of the missense variants was predicted using SIFT (http://www.fruitfly.org/seq_tools/splice.html) and Polyphen-2 (http://genetics.bwh.harvard.edu/pph2/). For an estimation of the evolutionary conservation of the amino acid sequence, we referred to the GERP++ score in the UCSC Genome Browser (http://genome.ucsc.edu/).

We considered the *CDH23* variants as potentially pathogenic when they satisfied the following criteria:

1. *CDH23* variants were not detected in the 200 normal control chromosomes (< 0.005) from our institute, which was a proposed ethnicity-specific MAF with a cut-off threshold (0.005) for autosomal recessive pathogenic variants [24, 25].
2. They were designated as ‘damaging or probably damaging’ by either SIFT or Polyphen-2, or a GERP++ score of higher than 3.
3. The residues of these variants were also conserved among several species.

A ‘probable DFNB12’ was defined as when we detected two potentially pathogenic *CDH23* variants in a *trans* configuration that fully satisfied the above criteria. In contrast, ‘possible DFNB12’ was defined as when we were able to identify only one *CDH23* variant whose pathogenic potential was previously documented.

We have deposited our whole sequencing data in our private SNUH-SNUBH sequencing database and have submitted the novel variants of *CDH23*, which were detected by next generation sequencing, to the Leiden Open Variation Database (LOVD) (http://databases.lovd.nl/shared/genes/CDH23).

### Comparison of ethnicity-specific MAF of the rare CDH23 variants among the SNHL cohort and the normal control cohort

To further evaluate the MAF of potentially pathogenic, rare *CDH23* variants in a larger size of the normal control population, a composite control cohort—which was comprised of up to 2726 normal Korean subjects (5452 alleles)—was used. The composite control cohort included 622 Korean Reference Genome (KRG) database (http://152.99.75.168/KRGDB), 700 Korean in-house exome data from the Korean National Institute of Health (KNIH), 1020 Korean control data from SGI, and 384 control individuals using TaqMan SNP Genotyping Assays (Applied Biosystems, Foster City, CA) **(S1 Fig)**.

**Phase I.** First, to evaluate the contribution of potentially pathogenic, rare *CDH23* variants to the Korean postlingual adult SNHL cohort, we compared the total frequency of potentially pathogenic, rare *CDH23* variants with MAF of less than 0.005 between our postlingual adult SNHL cohort and the control cohort from SGI (2040 alleles). Next, we genotyped the specific *CDH23* variants identified from our postlingual adult-onset SNHL cohort among the 1020 Korean control data from SGI (2040 alleles). We compared the MAF between the two groups using a Chi-square test.
**Phase II.** We tried to further calculate the MAF of *CDH23* variants detected from our postlingual adult SNHL cohort in this study and our previously reported pediatric SNHL cohort [9] in the ethnicity-matched composite control cohort (5452 alleles). Then we compared the MAF of *CDH23* variants from two cohorts among the composite control cohort using Fischer’s exact test and evaluated to see if there was any correlation between the MAF of *CDH23* variants and the clinical phenotype.

### Statistical analysis

Statistical analyses were performed using SPSS 18.0 (SPSS Inc., Chicago, USA). The level of statistical significance was defined as a p value of <0.05.

## Results

### Molecular genetic diagnosis and Clinical features of probands

**Fig 1:**
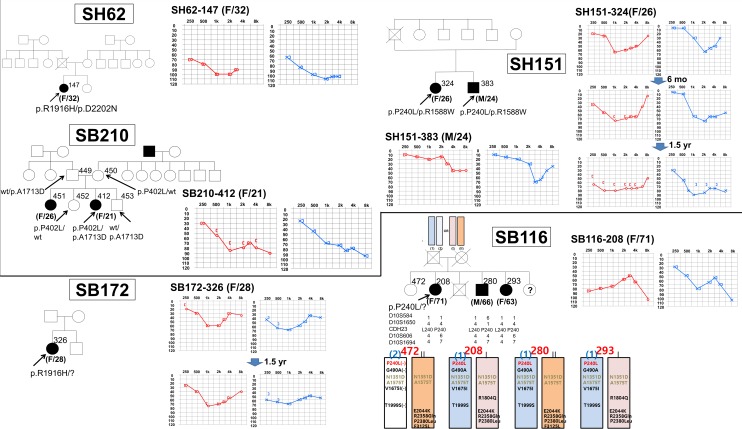
Pedigrees and audiograms of subjects from 5 families possibly carrying compound heterozygous *CDH23* mutations. Audiogram: Right (red) and left (blue) ear hearing thresholds. Pedigree: Filled symbols represent hearing-impaired individuals, and clear symbols denote those with normal hearing. Black arrow indicates the proband. Possible arrangement of *CDH23* variants in SB116 family based on haplotype and segregation study.

Among the 32 families of postlingual adult-onset NS-SNHL, with segregation in either a sporadic or AR fashion, we have identified four (12.5%) potential DFNB12 families: three probable DFNB12 families (SH62, SH151, and SB210) and one possible DFNB12 family (SB116). Notably, three of the four potential DFNB12 families segregated one definitely pathogenic DFNB12 variant in *trans* with a rare *CDH23* variant with unknown pathogenicity (SH62, SH151) or with a *CDH23* allele harboring a series of neighboring variants that were presumably minimally pathogenic when alone (SB116). The remaining family, SB210, co-segregated two rare *CDH23* variants of unknown pathogenicity with postlingual NS-SNHL **(Fig 1)**. The fifth family, SB172, carried only one potentially pathogenic *CDH23* variant (p.R1916H), precluding any conclusive molecular diagnosis of SB172 **(Fig 1)**.

Three families (SH62, SH151, and SB210) carrying two *CDH23* variants in a *trans* configuration manifested a rapidly progressive SNHL that started in their mid-teens to early twenties (**Table 1** and **Fig 1**). All of the affected subjects in these families underwent cochlear implantation (CI) or were scheduled to have CI in their twenties or early thirties.

**Table 1:** Genotype of individuals segregating homozygous or heterozygous mutations of *CDH23* identified by TRS or WES, and from the previous study by Kim et al. [9].

Patient	Sex/Age	Variant annotation	Location	Ref	Var	Coverage	Quality score	Found by
SH62-147	F/32	exon42:c.G5747A:p.R1916H	Chr10:73545422	G	A	178	99	TRS200
		exon46:c.G6604A:p.D2202N	Chr10:73553289	G	A	237	99	
SH151-324, 383	F/26	exon8:c.C719T:p.P240L	Chr10:73330641	C	T	193	60(Qcall)	TRS129
	M/23	exon37:c.C4762T:p.R1588W	Chr10:73501595	C	T	71	60(Qcall)	
SB210-412	F/21	exon12:c.C1205T:p.P402L	Chr10:73405652	C	T	59	60(Qcall)	TRS129
		exon38:c.C5138A:p.A1713D	Chr10:73538016	C	A	21	60(Qcall)	
SB116-208	F/71	exon8:c.C719T:p.P240L	Chr10:73330641	C	T	50	60(Qcall)	TRS129, WES
		Not determined						
SH59-133	F/3	exon8:c.C719T:p.P240L	Chr10:73330641	C	T	238	99	Kim et al. [9]
		exon37:c.C4853A:p.T1618K	Chr10:73537445	C	A	29	99	
SH97-211	F/1	exon8:c.C719T:p.P240L	Chr10:73330641	C	T	102	60(Qcall)	Kim et al. [9]
		exon8:c.C719T:p.P240L	Chr10:73330641	C	T	102	60(Qcall)	
SH164-359	F/1	exon8:c.C719T:p.P240L	Chr10:73330641	C	T	218	99	Kim et al. [9]
		exon8:c.C719T:p.P240L	Chr10:73330641	C	T	218	99	
SB56-103	F/4	exon8:c.C719T:p.P240L	Chr10:73330641	C	T	238	60(Qcall)	Kim et al. [9]
		exon58:c.8574delC:p.D2858EfsX8	Chr10:73567616	C	-	95	60(Qcall)	

In contrast, SB116-208, 280, and 293 showed a progressive, moderate SNHL that started in their sixties, much later than in three aforementioned probable DFNB12 families. In this family, WES was performed on all affected family members in SB-116 to exclude other causative genes. In this family, the pathogenic p.P240L variant of *CDH23* perfectly co-segregated with hearing loss phenotype, increasing the chance of *CDH23* compound heterozygote being the probable candidate etiology. However, a *CDH23* haplotype in *trans* with the p.P240L allele from one affected member (SB116-280) was different from those in the other two affected siblings (allele (II) vs (I) in **Fig 1**). In this family, the contribution of *CDH23* was not confirmed. We checked whether any variant from either *ATP2B2* or *PCDH15* can contribute to hearing loss in this family in *trans* with the p.P240L of *CDH23* as a modifier or in a digenic fashion, as previously suggested [14, 17, 26, 27]. However, none of the variants of *ATPB2* or *PCDH15* co-segregated with the hearing loss phenotype in this family (**S1 and S2 Tables**).

Lastly, one 28-year-old female (SB172-326) carrying only one potentially pathogenic *CDH23* variant (p.R1916H) also complained of bilateral progressive NS-SNHL which had started 2 years ago, and had started wearing bilateral hearing aids 1.5 years ago. Her recent audiogram demonstrated progressive, moderate SNHL on both sides, in which the hearing loss pattern was similar with SH151.

### Phase I: Contribution of *CDH23* variants to postlingual adult-onset sporadic or arSNHL

Potentially pathogenic *CDH23* variants were screened, both in our adult study and adult control cohorts. Eight alleles from six types of variants were detected: two p.P240L, one p.P402L, one p.R1588W, one p.A1713D, two p.R1916H, and one p.D2202N; the allele frequency of potentially pathogenic *CDH23* variants was determined to be 12.5% (95% CI, 4.4%–20.6%)(8/64) in our postlingual adult-onset sporadic or arSNHL cohort. However, the allele frequency of all potentially pathogenic *CDH23* variants in the 1020 ethnically-matched control WES data from Samsung Genome Institute (SGI) was calculated to be 5.53% (95% CI, 4.54%–6.52%) (113/2040) (**S3 Table**), which was significantly lower than that in our adult study cohort (*p* = 0.037 by Chi-square test) (**S1 Fig**).

Next, we focused on specific *CDH23* variants, which were identified from our postlingual adult-onset SNHL cohort among the 2040 alleles from the Korean control data provided by SGI. One of the control subjects carried a p.P240L variant, and the other control subject carried a p.R1588W variant, and there were no control subjects carrying any rare *CDH23* variants, such as p.R1916H, p.A1713D, p.P402L, or p.D2202N (described in **Table 2** and highlighted in **S3 Table**). Therefore, the allele frequency of these *CDH23* variants associated with postlingual adult-onset SNHL was calculated to be 0.098% (95% CI, 0%–0.23%)(2/2040) among the 1020 ethnicity-matched control subjects from SGI, which was significantly lower than that of our adult study cohort (*p*<0.0001 by Chi-square test) (**S1 Fig**).

**Table 2:** Ethnic-specific MAF and *in silico* pathogenicity prediction of *CDH23* variants in our study.

Variant	Patient	dbSNP	In-house exome from KNIH (n = 700)	KRG database (n = 622)	Genotyping (n = 384)	SGI (n = 1020)	Score in Pph2/ SIFT/ GERP	MAF in composite cohort	ExAC	1000 Genomes
p.T1618K (c.C4853A)	SH59-133 (Pediatric)	No data	0/1400	Not detected in unknown number	0/768	0/2040	Probably Damaging/ Damaging / 5.9	0% (0/4208)	N/A	N/A
p.P240L (c.C719T)	SH59-133 SH151-324 SB116-208 (Pediatric & Adult)	rs121908354 (flagged)				1/2040	Possibly Damaging/ Damaging/ 5.19	0.05% (1/2040)	0.00009	0.0002
p.D2202N (c.G6604A)	SH62-147 (Adult)	rs121908349 (flagged)	0/1400	Not detected in unknown number	0/768	0/2040	Probably Damaging/ Damaging/ 5.06	0% (0/4208)	0.000008	N/A
p.A1713D (c.C5138A)	SB210-412 (Adult)	No data				0/2040	Probably Damaging/ Damaging/ 5.4	0% (0/2040)	N/A	N/A
p.P402L (c.C1205T)	SB210-412 (Adult)	rs373168635 (Non-flagged)				0/2040	Probably Damaging/ Tolerated/ 4.91	0% (0/2040)	0.00003	N/A
p.R1588W (c.C4762T)	SH151-324 (Adult)	rs137937502	4/1400	2/1244	1/768	1/2040	Probably Damaging/ Damaging/ 3.24	0.15% (8/5452)	0.0002	0.0008
p.R1916H (c.G5747A)	SH62-147 (Adult)	rs746971522	3/1400	2/518	0/764	0/2040	Probably Damaging/ Damaging/ 4.28	0.11% (5/4722)	0.00006	N/A

KRG, Korean Reference Genome (http://152.99.75.168/KRGDB); ExAC, Exome Aggregation Consortium (http://exac.broadinstitute.org/); 1000 Genomes (https://www.ncbi.nlm.nih.gov/variation/tools/1000genomes/); N/A, not applicable

### Phase II: Comparison of MAF between *CDH23* variants detected in adult study cohort and pediatric study cohort among ethnicity-matched controls

Three adult patients (SH62-147, SH151-324, and SB210-412) carried two potentially pathogenic *CDH23* variants as a compound heterozygote, whereas four pediatric patients (SH59-133, SH97-211, SH164-359, and SB56-103) carried two *CDH23* variants either in homozygous or compound heterozygous state (**Table 1**) [9]. We focused on the pathogenicity of each *CDH23* allele, which were determined by their ethnic-specific MAF in our composite cohort (adult control cohort) **(S1 Fig)** [24, 25].

Ethnic-specific MAF of seven variants of *CDH23* in our adult and pediatric study cohorts was displayed **(Table 2)**. MAF of a missense variant, p.T1618K, of *CDH23*, which was detected from a pediatric cohort in *trans* configuration to p.P240L in SH59-133, was 0% (0/4208), while that of p.R1588W and p.R1916H, which were in *trans* to p.P240L and p.D2202N from adult patients, was 0.15% (95% CI, 0.05%–0.25%) (8/5452) and 0.11% (95% CI, 0.02%–0.2%) (5/4722), respectively, in our composite adult cohort (**Table 2**). The ethnic-specific MAF of p.T1618K detected from the pediatric SNHL population was significantly lower than that of p.R1588W and p.R1916H detected from the postlingual-onset adult SNHL population (*p* = 0.03, and 0.05 respectively by Fischer’s exact test). None of the control individuals carried p.D2202N, p.A1703D, and p.P402L.

## Discussion

Some *CDH23* mutations in humans have previously been reported to be associated with adult-onset postlingual progressive SNHL, in both Caucasians and Japanese [6, 14]. However, systematic documentation of the contribution of *CDH23* mutations to this late-onset postlingual progressive SNHL was not a main concern in these two reports. Instead, the role of a modifier gene, *ATP2B2*, was elucidated to account for the phenotypic differences among siblings [14]. Miyagawa et al. (2012) did not rigorously investigate the causal relationship between the *CDH23* genotype and its phenotype [6]. In contrast, our current study, which employs a genetic epidemiologic approach, clearly demonstrated that an alteration of the *CDH23* gene contributes to adult-onset postlingual progressive NS-SNHL. The rare *CDH23* alleles that satisfy our criteria for a potential pathogenicity were more frequently detected in Korean adult-onset postlingual progressive SNHL than in normal hearing controls, with statistical significance. In fact, this result is not surprising since the association of age-related progressive SNHL and some *Cdh23* alleles, such as *Cdh23*^ahl^ and *Cdh23*^erl^, has already been documented in mouse models with certain genetic backgrounds [17, 18, 28]. The replacement of a single nucleotide (A to G) in a *Cdh23* gene on progressive SNHL had been shown to prevent age-related SNHL phenotype [6, 17, 29, 30].

Based on our result, we could think that the major form of *CDH23-*related SNHL might be adult-onset progressive SNHL, rather than prelingual-onset severe-to-profound SNHL (DFNB12), at least in Koreans or East Asians. In fact, *CDH23* mutations accounted for 9.4% (3/32)—or possibly up to 12.5% (4/32)—of postlingual adult-onset SNHL, while the genetic load of *CDH23* mutations was 3.1% (4/128) in our pediatric cohort with prelingual-onset severe-to-profound SNHL [9]. A statistical analysis was performed to compare the frequency of *CDH23* mutation between the adult and pediatric hearing loss cohorts; a difference was shown with marginal significance (*p* = 0.051 by Fischer’s exact test). From this observation, we can assert that *CDH23* variants might contribute more to adult-onset progressive SNHL than to prelingual-onset severe-to-profound SNHL. However, we need to be cautious to draw a firm conclusion from these results due to the relatively small number of subjects in this study.

One important finding from our study is that the p.P240L allele of *CDH23*, which turned out to be the founder allele among the prelingual DFNB12 Korean subjects, was revisited in our adult-onset postlingual SNHL cohort. Two (SH151 and SB116) of the five families with co-segregating adult-onset progressive SNHL with at least one potentially pathogenic *CDH23* variant turned out to have the p.P240L allele. A contribution of the p.P240L allele to our adult-onset postlingual SNHL was confirmed by a higher frequency of this allele (2/64) in our adult SNHL cohort than in normal controls (1/2040) (*p*<0.0001 by Fischer’s exact test). The p.P240L homozygotes were previously reported to cause prelingual severe-to-profound SNHL in a majority of cases [6, 9, 31]. According to our study, as well as previous Japanese studies, audiological phenotypes of *CDH23* compound heterozygotes that carry one p.P240L allele seem to be highly variable [6, 31]. Indeed, SH151-324 carrying p.P240L/p.R1588W manifested progressive SNHL, which started from mid to high frequencies at the age of 20. In contrast, SB116-208 showed progressive SNHL that became noticeable after the age of 60 years in the present study. Given this, it can be postulated that auditory phenotypes of these families depend on the pathogenic potential or residual CDH23 protein dosage from the *trans CDH23* allele to p.P240L. In our previous study with a pediatric pre-lingual SNHL population, the *trans* allele of p.P240L was p.P240L itself, p.D2858E*fs*X8, which was a truncation mutation, and p.T1618K, respectively. It is easily conceivable that p.D2858E*fs*X8 leads to a serious deleterious effect on proteins. The MAF of this missense variant, p.T1618K, was extremely low as indicated by zero detection among 4208 control alleles, implying a strong pathogenic potential [32]. Among the two postlingual SNHL families (SH151 and SB116) carrying p.P240L, the *trans* allele of p.P240L of *CDH23* from SH151-324 was p.R1588W; however, none of the potential polymorphisms of *CDH23* in *trans* with p.P240L was compatible with the segregation of SNHL in SB116. The pathogenic potential of p.R1588W of *CDH23* was previously disputed due to the presence of normal hearing from one homozygous carrier of p.R1588W [6]. However, it is likely that p.R1588W, presumably with a mild pathogenicity, can exert its pathogenic effect leading to progressive postlingual SNHL when in *trans* with a strongly pathogenic p.P240L.

The role of *CDH23* mutations in SB116 is enigmatic. The *CDH23* haplotype of *trans* allele to p.P240L was not shared by all the siblings with SNHL in this family. This may suggest that mutations in *CDH23* do not account for SNHL in SB116 and that the detection of p.P240L was fortuitous. However, some of the frequent neighboring single nucleotide polymorphisms of *CDH23* were indeed shared en bloc by all three siblings with SNHL **(Fig 1)**. The collective effects of these SNPs in *CDH23* might exert a pathogenic effect, albeit not severe, in a *trans* configuration with p.P240L, leading to progressive SNHL prominent after the 60’s. A perfect co-segregation of p.P240L of *CDH23* with the SNHL phenotype in SB116 supported this hypothesis. Significantly late onset age of SNHL in SB116 might also increase the possibility of a relationship between *CDH23* and presbycusis in an older population.

Differential auditory phenotype, depending on the combination of the two *Cdh23* alleles in mice, showed a striking resemblance to our observation. Homozygous mice carrying the functionally null mutations of *Cdh23*, such as *Cdh23*^v^ or *Cdh23*^v-ngt^, manifest congenital profound SNHL and a severe vestibular phenotype [33, 34]. In contrast, homozygosity with respect to *Cdh23*^753A^ (hypomorphic Ahl allele) leads to increased susceptibility to age-related SNHL, thereby showing severe hearing loss by 9–12 months of age, under certain genetic backgrounds [18]. Interestingly, the compound heterozygous mice with one null allele of *Cdh23*^v-ngt^ and one hypomorphic allele (*Cdh*23^*ahl*^*)* under C57BL/6J showed an intermediate phenotype: hearing impairment that started from the age of 4 months which increased in severity in an age-dependent manner [35]. Taken together, the combination of two *Cdh23* alleles with a different pathogenic potential decided the fate of auditory phenotype in mice.

From this perspective, we looked into the human *CDH23* variants that we detected from our present study, as well as from our previous study by Kim et al. (2015) [9]. First, we made a comparison of the ethnic-specific MAF between the missense variants detected from our adult SNHL cohort and the missense variants from our pediatric SNHL cohort. We observed significantly higher MAF of p.R1588W and p.R1916H from the postlingual-onset adult SNHL population than that of p.T1618K, which was in *trans* with p.P240L [9] in a pre-lingual profound SNHL. The most likely explanation would be that the difference in the pathogenic toxicity of the two alleles (p.R1588W vs p.T1618K) in *trans* with the known pathogenic allele, p.P240L, decided the fate of auditory phenotype in two subjects. The milder pathogenic potential of p.R1588W as compared with p.T1618K could be indirectly supported by normal hearing from the p.R1588W homozygous carrier [6] and also by the higher Korean MAF [32]. Another two missense variants, p.D2202N (rs121908349 flagged) and p.A1713D, in postlingual adult-onset SNHL, which were detected in *trans* with p.R1916H and p.P402L, respectively, may exert just as strong pathogenic potential as p.P240L, especially considering the GERP score of more than 5, in *silico* prediction results and extremely low MAF **(Table 2).** Additionally, the pathogenic potential of p.D2202N was previously described [1]. In contrast, p.R1916H and p.P402L (rs373168635) may also serve as milder pathogenic alleles, considering their relatively low GERP score of <5, MAF, and in *sili*co prediction results **(Table 2)**. From this explanation, it is possible to suggest MAF as a predictor of the clinical fate of DFNB12.

**Fig 2:**
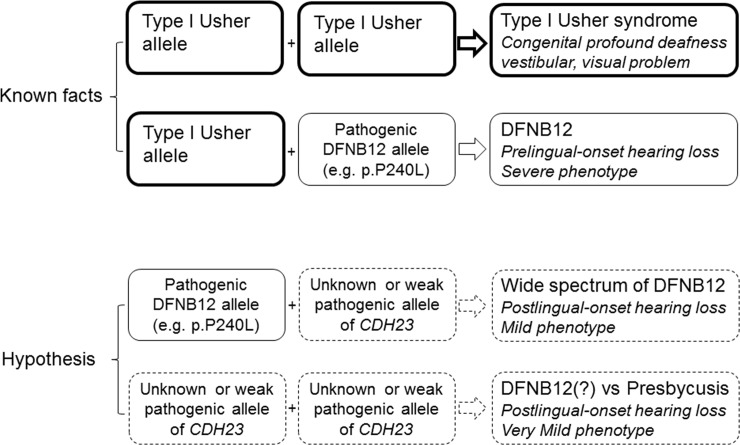
Suggestion of Genotypic Hierarchy of *CDH23* mutations deciding the fate of USH1D and DFNB12.

In a previous genotype-phenotype correlation study, it was suggested that the phenotypic consequence of the compound heterozygosity for a DFNB12 allele in *trans* configuration with a predicted USH1D allele of *CDH23* was determined, mostly by the DFNB12 allele [5]. In this present study, we tried to extend this hypothesis to include the phenotypic consequence of the compound heterozygosity of a known pathogenic DFNB12 allele in *trans* configuration with *CDH23* allele with an unknown pathogenicity, or to even include the compound heterozygosity of two *CDH23* alleles with an unknown pathogenicity **(Fig 2)**.

## Conclusions

This study shows an important contribution of *CDH23* variants to postlingual NS-SNHL. It also broadens the phenotypic spectrum of DFNB12 to include even presbycusis depending on the allelic combinations of *CDH23*. We also propose a potential role of Korean MAF of rare *CDH23* variants as a predictor of pathogenic potential and clinical fate of DFNB12 in postlingual NS-SNHL. These patients carrying such *CDH23* variants need to be counseled about cochlear implantation, based on the nature of rapid progression to severe-to-profound SNHL irrespective of age at diagnosis. Furthermore, since it is possible to correct age-related hearing loss in mice by repairing a single mutation in the *Cdh23* allele, it may be feasible to treat *CDH23-*related hearing loss in humans with gene therapy in the near future [29].

## Supporting Information

S1 Fig Flow chart of the study combining Phase I and II. Phase I: Comparison of carrier frequency of potentially pathogenic *CDH23* alleles between our postlingual adult SNHL cohort and the control cohort. Phase II: Comparison of MAF between *CDH23* variants detected in postlingual adult SNHL. cohort and prelingual pediatric SNHL cohort among the composite control cohort.
(TIF)

S1 Table lists the screening result of *ATP2B2* variants in SB116. (DOCX)

S2 Table lists the screening result of *PCDH15* variants in SB116. (DOCX)

S3 Table lists potentially pathogenic *CDH23* variants in 1020 ethnicity-matched control WES data from SGI. (DOCX)
