# Supplementary material for: Discovery of CDH23 as a Significant Contributor to Progressive Postlingual Sensorineural Hearing Loss in Koreans
Source: PLoS One. 2016 Oct 28;11(10):e0165680. doi: 10.1371/journal.pone.0165680 (PMC5085094; doi:10.1371/journal.pone.0165680)
Supplement: S1 Table — (DOCX) [file pone.0165680.s002.docx]

**S1 Table**. Screening result of *ATP2B2* variants in SB116

| **Exonic Func** | **Genbank ID** | **Exon** | **Nucleotide** | **AA** | **Chr** | **Start** | **End** | **Ref** | **Alt** | **1000G** | **dbSNP137** | **Frequency in SGI normal reference** | **INFO AC** | **INFO AF** | **SB116-208** | **SB116-**  **280** | **SB116-**  **293** |
| --- | --- | --- | --- | --- | --- | --- | --- | --- | --- | --- | --- | --- | --- | --- | --- | --- | --- |
| synonymous SNV | NM_001683 | exon19 | c.G3222A | p.A1074A | chr3 | 10379923 | 10379923 | C | T | 0.53 | rs35678 (NonFlagged) | 0.622396 | 4 | 0.667 | hetero | homo | hetero |
| synonymous SNV | NM_001683 | exon17 | c.C2808T | p.S936S | chr3 | 10382363 | 10382363 | G | A | NA | N/A | 0.005208 | 3 | 0.5 | hetero | hetero | hetero |
| synonymous SNV | NM_001683 | exon7 | c.A915G | p.Q305Q | chr3 | 10420087 | 10420087 | T | C | 0.45 | rs751122 (NonFlagged) | 0.627604 | 3 | 0.5 | hetero | hetero | hetero |

Chr: chromosome; AA: Amino Acid; INFO AC: Allele Count; INFO AF: Allele Frequency; SGI: Samsung Genome Institute; N/A: not applicable
