# Supplementary material for: Discovery of CDH23 as a Significant Contributor to Progressive Postlingual Sensorineural Hearing Loss in Koreans
Source: PLoS One. 2016 Oct 28;11(10):e0165680. doi: 10.1371/journal.pone.0165680 (PMC5085094; doi:10.1371/journal.pone.0165680)
Supplement: S2 Table — (DOCX) [file pone.0165680.s003.docx]

**S2 Table**. Screening result of *PCDH15* variants in SB116

| **Exonic Func** | **Genbank ID** | **Exon** | **Nucleotide** | **AA** | **Chr** | **Start** | **End** | **Ref** | **Alt** | **1000G** | **dbSNP137** | **Frequency in SGI normal reference** | **INFO AC** | **INFO AF** | **SB116-208** | **SB116-**  **280** | **SB116-**  **293** |
| --- | --- | --- | --- | --- | --- | --- | --- | --- | --- | --- | --- | --- | --- | --- | --- | --- | --- |
| nonframeshift deletion | NM_001142767 | Exon31 | c.5167_5172del | p.1723_1724del | Chr10 | 55582194 | 55582199 | AGGAGC | - | N/A | N/A | 0.015625 | 2 | 0.333 | hetero | No Variant | hetero |
| synonymous  SNV | NM_001142767 | Exon31 | c.C4461A | p.P1487P | Chr10 | 55582905 | 55582905 | G | T | 0.29 | rs10825114  (NonFlagged) | 0.78125 | 4 | 0.667 | hetero | homo | hetero |
| nonsynonymous SNV | NM_001142765 | Exon20 | c.C2671T | p.R891C | Chr10 | 55721637 | 55721637 | G | A | 0.0014 | rs201816080  (NonFlagged) | 0.013020833 | 2 | 0.333 | hetero | No Variant | hetero |
| nonsynonymous SNV | NM_001142765 | Exon19 | c.G2573A | p.R858Q | Chr10 | 55755491 | 55755491 | C | T | 0.3 | rs2135720  (NonFlagged) | 0.505208333 | 4 | 0.667 | homo | No Variant | homo |
| nonsynonymous SNV | NM_001142767 | Exon10 | c.A1193C | p.D398A | Chr10 | 55955444 | 55955444 | T | G | 0.34 | rs4935502  (NonFlagged) | 0.872395833 | 5 | 0.833 | homo | hetero | homo |
| synonymous SNV | NM_001142767 | Exon10 | c.T1152C | p.T384T | Chr10 | 55955485 | 55955485 | A | G | 0.22 | rs7921598  (NonFlagged) | 0.059895833 | 1 | 0.167 | No Variant | hetero | No Variant |
| nonsynonymous SNV | NM_001142767 | Exon10 | c.G1027A | p.G343S | Chr10 | 55955610 | 55955610 | C | T | 0.21 | rs10825269  (NonFlagged) | 0.059895833 | 1 | 0.167 | No Variant | hetero | No Variant |

Chr: chromosome; AA: Amino Acid; INFO AC: Allele Count; INFO AF: Allele Frequency; SGI: Samsung Genome Institute; N/A: not applicable
