# Supplementary material for: Discovery of CDH23 as a Significant Contributor to Progressive Postlingual Sensorineural Hearing Loss in Koreans
Source: PLoS One. 2016 Oct 28;11(10):e0165680. doi: 10.1371/journal.pone.0165680 (PMC5085094; doi:10.1371/journal.pone.0165680)
Supplement: S3 Table — (DOCX) [file pone.0165680.s004.docx]

**S3 Table**. Potentially pathogenic *CDH23* variants in 1020 ethnicity-matched control WES data from SGI

| Chr | Start | End | Ref | Alt | Func | Exonic Func | MAF | Allele No. | GERP++ | SIFT | PolyPhen2 HVAR |
| --- | --- | --- | --- | --- | --- | --- | --- | --- | --- | --- | --- |
| 10 | 73199596 | 73199596 | G | A | exonic | nonsynonymous SNV | 0.001965 | 4 | 3.67 | 0.31,0.69,T | 0.0,B |
| 10 | 73270926 | 73270926 | C | T | exonic | nonsynonymous SNV | 0.000491 | 1 | 5.58 | 0.12,0.88,T | 0.943,D |
| **10** | **73330641** | **73330641** | **C** | **T** | **exonic** | **nonsynonymous SNV** | **0.000491** | **1** | **5.19** | **0.23,0.77,T** | **0.999,D** |
| 10 | 73375302 | 73375302 | G | C | exonic | nonsynonymous SNV | 0.000491 | 1 | 4.22 | 0.4,0.60,T | 0.053,B |
| 10 | 73376998 | 73376998 | G | A | exonic | nonsynonymous SNV | 0.000491 | 1 | 5.47 | 0.34,0.66,T | 0.996,D |
| 10 | 73405681 | 73405681 | C | T | exonic | nonsynonymous SNV | 0.000491 | 1 | 4.91 | 0.1,0.90,T | 0.997,D |
| 10 | 73405717 | 73405717 | G | A | exonic | nonsynonymous SNV | 0.000982 | 2 | 3.99 | 0.1,0.90,T | 0.952,D |
| 10 | 73406267 | 73406267 | A | G | exonic | nonsynonymous SNV | 0.000491 | 1 | 5.69 | 0.21,0.79,T | 0.978,D |
| 10 | 73434867 | 73434867 | A | C | splicing | N/A | 0.000491 | 1 | 5.72 | N/A | N/A |
| 10 | 73437293 | 73437293 | C | T | exonic | nonsynonymous SNV | 0.000491 | 1 | 5.51 | 0.02,0.98,D | 0.999,D |
| 10 | 73442277 | 73442277 | A | G | exonic | nonsynonymous SNV | 0.000491 | 1 | 5.31 | 0,1.00,D | 1.0,D |
| 10 | 73450293 | 73450293 | A | G | exonic | nonsynonymous SNV | 0.000491 | 1 | 5.12 | 0.17,0.83,T | 0.989,D |
| 10 | 73453963 | 73453963 | G | A | exonic | nonsynonymous SNV | 0.000982 | 2 | 5.55 | 0.27,0.73,T | 0.832,P |
| 10 | 73454011 | 73454011 | G | A | exonic | nonsynonymous SNV | 0.000491 | 1 | 5.55 | 0.33,0.67,T | 0.766,P |
| 10 | 73464753 | 73464753 | T | C | exonic | nonsynonymous SNV | 0.000491 | 1 | 5.5 | 0,1.00,D | 0.854,P |
| 10 | 73464788 | 73464788 | G | A | exonic | nonsynonymous SNV | 0.000982 | 2 | 5.5 | 0.83,0.17,T | 0.09,B |
| 10 | 73466702 | 73466702 | A | G | exonic | nonsynonymous SNV | 0.000982 | 2 | 5.28 | 0.61,0.39,T | 0.633,P |
| 10 | 73466738 | 73466738 | G | A | exonic | nonsynonymous SNV | 0.000982 | 2 | 5.28 | 0.59,0.41,T | 0.166,B |
| 10 | 73468921 | 73468921 | T | C | exonic | nonsynonymous SNV | 0.000491 | 1 | 4.97 | 0,1.00,D | 1.0,D |
| 10 | 73468926 | 73468926 | C | T | exonic | nonsynonymous SNV | 0.000982 | 2 | 2.84 | 0.03,0.97,D | 1.0,D |
| 10 | 73472463 | 73472463 | G | A | exonic | nonsynonymous SNV | 0.001473 | 3 | 4.01 | 0,1.00,D | 0.963,D |
| 10 | 73472497 | 73472497 | G | A | exonic | nonsynonymous SNV | 0.000491 | 1 | 4.92 | 0.36,0.64,T | 0.636,P |
| 10 | 73472553 | 73472553 | G | A | exonic | nonsynonymous SNV | 0.002947 | 4 | 4.92 | 0.09,0.91,T | 0.21,B |
| 10 | 73483829 | 73483829 | G | A | exonic | nonsynonymous SNV | 0.002599 | 4 | 4.89 | 0.66,0.34,T | 0.164,B |
| 10 | 73485221 | 73485221 | G | T | exonic | nonsynonymous SNV | 0.000491 | 1 | 4.23 | 0.33,0.67,T | 0.231,B |
| 10 | 73492014 | 73492014 | G | A | exonic | nonsynonymous SNV | 0.000491 | 1 | 4.81 | 0,1.00,D | 0.994,D |
| 10 | 73492131 | 73492131 | C | T | exonic | nonsynonymous SNV | 0.000491 | 1 | 4.99 | 0,1.00,D | 0.999,D |
| 10 | 73494027 | 73494027 | C | T | exonic | nonsynonymous SNV | 0.001965 | 4 | 0.907 | 0.02,0.98,D | 1.0,D |
| 10 | 73498261 | 73498261 | A | G | exonic | nonsynonymous SNV | 0.000982 | 2 | 4.12 | 0.52,0.48,T | 0.029,B |
| 10 | 73498294 | 73498294 | C | T | exonic | nonsynonymous SNV | 0.001965 | 4 | 4.39 | 0.03,0.97,D | 0.453,P |
| 10 | 73498391 | 73498391 | G | A | exonic | nonsynonymous SNV | 0.001473 | 2 | 5.38 | 0.29,0.71,T | 0.998,D |
| 10 | 73499432 | 73499432 | C | T | exonic | nonsynonymous SNV | 0.000491 | 1 | 3.73 | 0.28,0.72,T | 0.006,B |
| 10 | 73501520 | 73501520 | C | A | exonic | nonsynonymous SNV | 0.000491 | 1 | 3.95 | 0.16,0.84,T | 0.998,D |
| **10** | **73501595** | **73501595** | **C** | **T** | **exonic** | **nonsynonymous SNV** | **0.000491** | **1** | **3.24** | **0.02,0.98,D** | **0.996,D** |
| 10 | 73501658 | 73501658 | G | A | exonic | nonsynonymous SNV | 0.000983 | 2 | 5.09 | 0.86,0.14,T | 0.856,P |
| 10 | 73538009 | 73538009 | G | A | exonic | nonsynonymous SNV | 0.001473 | 2 | 3.57 | 0.2,0.80,T | 0.998,D |
| 10 | 73544766 | 73544766 | T | G | exonic | nonsynonymous SNV | 0.000982 | 2 | 3.17 | 0.66,0.34,T | 0.072,B |
| 10 | 73544772 | 73544772 | G | A | exonic | nonsynonymous SNV | 0.000491 | 1 | 4.41 | 0.29,0.71,T | 0.497,P |
| 10 | 73545485 | 73545485 | T | G | exonic | nonsynonymous SNV | 0.000491 | 1 | 4.05 | 0.08,0.92,T | 0.776,P |
| 10 | 73550910 | 73550910 | G | T | exonic | nonsynonymous SNV | 0.000491 | 1 | 3.98 | 0.09,0.91,T | 0.937,D |
| 10 | 73553028 | 73553028 | C | A | exonic | nonsynonymous SNV | 0.000491 | 1 | 5.51 | 0.45,0.55,T | 0.962,D |
| 10 | 73553214 | 73553214 | C | G | exonic | nonsynonymous SNV | 0.000491 | 1 | 5.28 | 0.4,0.60,T | 0.599,P |
| 10 | 73556860 | 73556860 | G | A | splicing | N/A | 0.001473 | 3 | 5.87 | N/A | N/A |
| 10 | 73558192 | 73558192 | G | A | exonic | nonsynonymous SNV | 0.001473 | 3 | 3.68 | 0.49,0.51,T | 0.009,B |
| 10 | 73558958 | 73558958 | G | A | exonic | nonsynonymous SNV | 0.001473 | 3 | 5.55 | 0.34,0.66,T | 0.992,D |
| 10 | 73559385 | 73559385 | C | T | exonic | nonsynonymous SNV | 0.000491 | 1 | 5.55 | 0,1.00,D | 0.997,D |
| 10 | 73562763 | 73562763 | A | G | exonic | nonsynonymous SNV | 0.000491 | 1 | 5.02 | 1,0.00,T | 0.015,B |
| 10 | 73567321 | 73567321 | T | C | exonic | nonsynonymous SNV | 0.000491 | 1 | 4.4 | 0.18,0.82,T | 0.633,P |
| 10 | 73567358 | 73567358 | C | G | exonic | nonsynonymous SNV | 0.002456 | 5 | 5.61 | 0.22,0.78,T | 0.151,B |
| 10 | 73567687 | 73567687 | T | A | exonic | nonsynonymous SNV | 0.001473 | 3 | 4.34 | 0.84,0.16,T | 0.011,B |
| 10 | 73569678 | 73569678 | G | A | exonic | nonsynonymous SNV | 0.000491 | 1 | 5.61 | 0.43,0.57,T | 0.369,B |
| 10 | 73569682 | 73569682 | C | T | exonic | nonsynonymous SNV | 0.000982 | 2 | 5.61 | 0.07,0.93,T | 0.621,P |
| 10 | 73571147 | 73571147 | G | C | exonic | nonsynonymous SNV | 0.000982 | 2 | 4.6 | 0.02,0.98,D | 0.235,B |
| 10 | 73571307 | 73571307 | G | A | exonic | nonsynonymous SNV | 0.000982 | 2 | 4.46 | 0.02,0.98,D | 0.002,B |
| 10 | 73571735 | 73571735 | A | G | exonic | nonsynonymous SNV | 0.000491 | 1 | 5.82 | 0.04,0.96,D | 0.002,B |
| 10 | 73572268 | 73572268 | C | T | exonic | nonsynonymous SNV | 0.000491 | 1 | 5.54 | 0,1.00,D | 0.999,D |
| 10 | 73574829 | 73574829 | G | A | exonic | nonsynonymous SNV | 0.001967 | 4 | 5.14 | 0.02,0.98,D | 0.999,D |
| 10 | 73574899 | 73574899 | G | A | exonic | nonsynonymous SNV | 0.001473 | 3 | 5.14 | 0,1.00,D | 0.995,D |
| 10 | 73574992 | 73574992 | G | A | exonic | nonsynonymous SNV | 0.000989 | 2 | 5.42 | 0.03,0.97,D | 0.791,P |
| 10 | 73574997 | 73574997 | G | A | exonic | nonsynonymous SNV | 0.000992 | 2 | 5.42 | 0.01,0.99,D | 0.994,D |
| 10 | 73575030 | 73575030 | C | A | exonic | nonsynonymous SNV | 0.001517 | 3 | 3.23 | 0,1.00,D | 0.996,D |

Chr, chromosome; MAF, minor allele frequency

SIFT: D, damaging; T, Tolerated

Polyphen2: D, probably damaging; P, possibly damaging; B, benign
